# The role of electrocardiogram in the diagnosis of dextrocardia with mirror image atrial arrangement and ventricular position in a young adult Nigerian in Ile-Ife: a case report

**DOI:** 10.1186/s13256-015-0695-4

**Published:** 2015-09-28

**Authors:** Oluwadare Ogunlade, Abiodun O. Ayoka, Rufus O. Akomolafe, Olumide S. Akinsomisoye, Adedayo I. Irinoye, Adewale Ajao, Muritala A. Asafa

**Affiliations:** 1 Department of Physiological Sciences, Obafemi Awolowo University, Ile-Ife, Nigeria; 2 Medical and Health Services, Obafemi Awolowo University, Ile-Ife, Nigeria

**Keywords:** Dextrocardia, Electrocardiogram, Nigerian, Young adult

## Abstract

**Introduction:**

Dextrocardia with situs inversus is a rare congenital disease. In patients with this condition, the heart is presented as a mirror image of itself with its apex pointing to the right. The pulmonary and abdominal anatomies are reversed. Dextrocardia with situs inversus occurs at birth but its diagnosis may be in adulthood. This case advances knowledge by graphically describing the unusual electrocardiographic features of dextrocardia in a young adult.

**Case presentation:**

We report a case of a 22-year-old Nigerian man of Yoruba ethnicity who presented himself for preadmission medical test. He had a standard 12-lead electrocardiogram which revealed uncommon features: inversion of P waves in leads I, aVL and aVR; dominantly negative QRS waves in leads I, V1 to V6; reverse R wave progression in chest leads; low voltage in V4 to V6; extreme QRS axis; flattened T waves in V4 to V6 and aVR; and inverted T waves in lead I and aVL. An electrocardiogram diagnosis of dextrocardia was made. The differential diagnosis considered was right ventricular hypertrophy. A cardiovascular examination showed pulse rate of 70 beats per minute, blood pressure of 119/62mmHg, visible cardiac impulse at right precordium, apex beat was located at his fifth right intercostal space mid-clavicular line. A chest X-ray (posterior anterior view) including upper abdomen showed dextrocardia; his aortic arch was located on the right. His stomach bubble was located below his right hemidiaphragm. His trachea was slightly deviated to the left. The findings in his lung fields were not remarkable. Abdominopelvic ultrasonography showed that right-sided intra-abdominal organs (liver, gallbladder) were located on the left while left-sided organs (stomach, spleen) were located on the right. His abdominal aorta was on the right while his inferior vena cava was located on the left. A diagnosis of dextrocardia with situs inversus was made ultrasonographically.

**Conclusions:**

A properly interpreted electrocardiogram was useful in suspecting the diagnosis of dextrocardia with situs inversus. So, an analysis of a relatively simple and non-invasive diagnostic tool such as an electrocardiogram allows for suspicion of a cardiovascular anomaly in a setting of scarce diagnostic resources.

## Introduction

Dextrocardia with situs inversus is an anomaly. Hieronymus Fabricius was credited with the first description of situs inversus in 1606 while Marco Aurelio Severino (1580–1656), an Italian surgeon and anatomist, described dextrocardia in 1643 [1]. In patients with this complex medical condition, the heart is malrotated and presented as a mirror image of itself with its apex pointing to the right side of the body. The morphologic right atrium is on the left and the morphologic left atrium is on the right. The normal pulmonary anatomy is reversed with the left lung having three lobes and the right lung having two lobes. The right-sided abdominal organs, liver and gallbladder are located on the left whereas the spleen and stomach are located on the right [2]. Dextrocardia with situs inversus occurs at birth but its diagnosis may be in adulthood. The diagnosis can be made through clinical evaluation, electrocardiogram (ECG), chest X-ray and abdominal ultrasonography, echocardiography and computed tomography (CT) [3–5]. In a few cases, diagnoses were made at autopsy or during dissection of cadavers [6, 7]. This case illustrates the role of an ECG in the diagnosis of dextrocardia. It advances knowledge by graphically describing the unusual ECG features of dextrocardia in a young adult.

## Case presentation

**Fig. 1:**
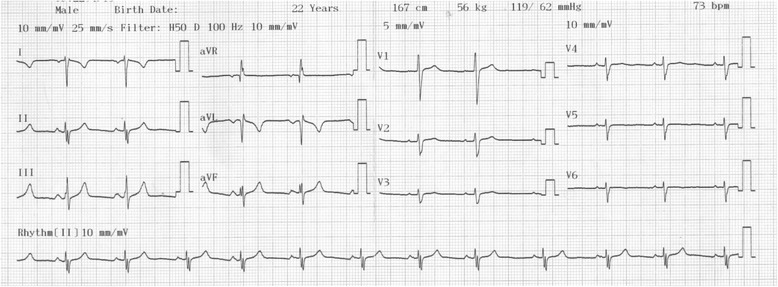
A resting standard 12-lead electrocardiogram of a 22-year-old Nigerian man. He had a heart rate of 73 beats per minute, inversion of P waves in leads I, aVL and aVR, dominant S waves in leads I and V1 to V6, reversed R wave progression in chest leads, low voltage QRS axis in V4 to V6, extreme QRS axis, flattened T waves in V4 to V6 and aVR and inverted T waves in lead I and aVL

**Fig. 2:**
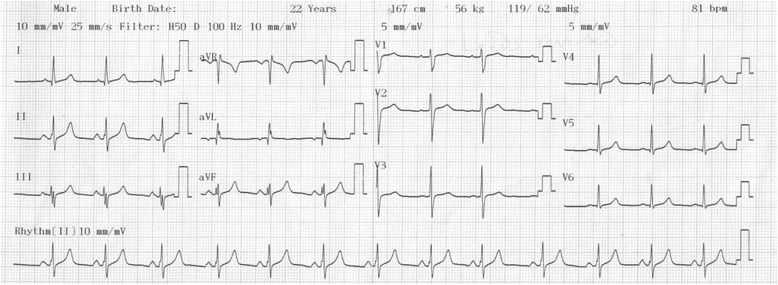
A resting standard 12-lead electrocardiogram of a 22-year-old Nigerian man with the electrocardiogram electrodes reversed. The electrocardiogram electrodes were reversed by placing the chest electrodes in a mirror image position on the right side of his chest and reversing the left and right limb leads. All the previous changes (Fig. 1) were reversed and a normal electrocardiographic pattern of a young adult man occurred

**Fig. 3:**
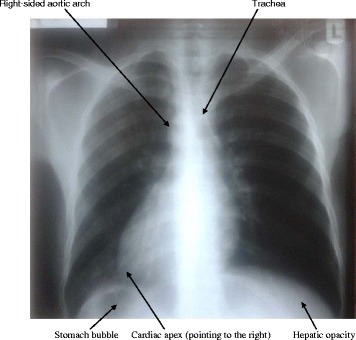
Chest X-ray (posterior anterior view) of a 22-year-old Nigerian man showing dextrocardia, with the cardiac apex pointing to the right. His aortic arch was located on the right. His stomach bubble was located below his right hemidiaphragm. His hepatic opacity was located below his left hemidiaphragm. His trachea was slightly deviated to the left

We report a case of a 22-year-old Nigerian man of Yoruba ethnicity who presented himself for electrocardiographic screening as a pre-admission medical test. He had a standard 12-lead ECG which revealed uncommon features: inversion of P waves (atrial depolarization) in leads I, aVL and aVR; dominant S waves in leads I, V1 to V6 with reversed R wave progression in chest leads; low voltage QRS axis in V4 to V6; extreme QRS axis; flattened T waves (ventricular repolarization) in V4 to V6 and aVR; and inverted T waves in lead I and aVL (Fig. 1). An ECG diagnosis of dextrocardia was made; the differential diagnosis considered was right ventricular hypertrophy. The ECG electrodes were then placed in reverse order (mirror image position) on his body, which produced a normal standard 12-lead ECG pattern of a young adult. This confirmed dextrocardia with mirror image atrial arrangement (Fig. 2). On further evaluation, he revealed a 5-year history of recurrent abdominal pain and a 3-week history of catarrh, fever, cough, exercise intolerance and progressive weight loss. There was no history of chest pain, orthopnea, paroxysmal nocturnal dyspnea, palpitation or body swelling. He had no history of contact with someone with a chronic cough. There was a positive history of twins (twice) in his nuclear family. A physical examination revealed a slim young man not in obvious respiratory distress; he was not cyanosed, pale or febrile. He had neither finger clubbing nor pedal edema. A cardiovascular examination showed a pulse rate of 70 beats per minute, blood pressure of 119/62mmHg, visible cardiac impulse at right precordium, apex beat was located at his fifth right intercostal space midclavicular line, and first and second heart sounds were heard. A chest X-ray (posterior anterior view) including upper abdomen showed dextrocardia, with the cardiac apex pointing to the right. His aortic arch was located on the right. His stomach bubble was located below his right hemidiaphragm. His hepatic opacity was located below his left hemidiaphragm. His trachea was slightly deviated to the left. The findings in the lung fields were not remarkable (Fig. 3). An abdominopelvic ultrasonography showed that his liver was located on the left side; it measured 14.5cm in span. The liver margin was smooth and the intrahepatic ducts and vascular channels were preserved. The gallbladder was seen in its inferior border. His spleen was located in the right hypochondrium; it was preserved sonographically. His stomach was on the right and his duodenum was located on the left. His two kidneys were seen in the renal beds bilaterally. His abdominal aorta was on the right while his inferior vena cava was located on the left. His urinary bladder was centrally placed and an assessment of situs inversus was made ultrasonographically. Overall, the diagnosis of dextrocardia with situs inversus to exclude Kartagener syndrome was made. Sputum microscopy, culture and sensitivity (MCS), sputum for acid-fast bacilli, chest CT scan and echocardiography were requested. He was placed on a combination of amoxycillin and azithromycin pending the result of his sputum tests. His sputum MCS showed *Klebsiella* species sensitive to cefuroxime. The sputum acid-fast bacilli test was negative. He was scheduled for echocardiography to determine cardiac structure and function; he was also scheduled for a chest CT. However, he had financial challenges which delayed completion of the investigations. Following completion of the initial antibiotics dosages, he was placed on cefuroxime and he became symptom free within 2 weeks of treatment.

## Discussion

**Fig. 4:**
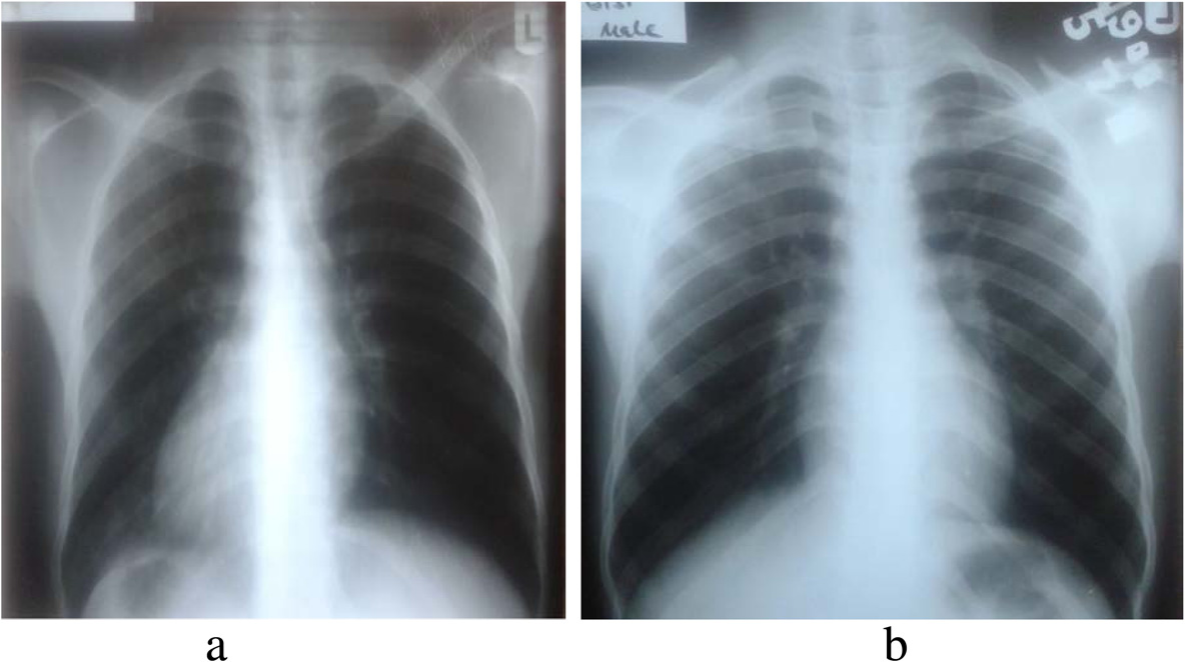
Chest X-ray of a 22-year-old Nigerian man with dextrocardia and situs inversus (**a**) compared with a chest X-ray of a 20-year-old Nigerian man (**b**) with the heart and abdominal organs normally positioned

An ECG is a graphical record of the electrical activities of a heart obtained on the body surface. It is a basic non-invasive investigation with great application in medical practice [8]. ECG is a cheap, portable and harmless medical test that is very useful in the assessment of cardiac electrical activities and to some extent cardiac structures. ECG was acknowledged as the most commonly conducted cardiovascular diagnostic procedure in clinical practice [9]. Dextrocardia is one of the cardiac anomalies that presents itself with unusual and specific electrocardiographic features which include inversion of P waves in leads I and aVL, dominant S waves in leads I and V1 to V6, reversed R wave progression in chest leads, low voltage QRS axis in V4 to V6, extreme QRS axis, flattened T waves in V4 to V6 and aVR and inverted T waves in lead I and aVL. These findings were present in our patient and were consistent with ECG findings of dextrocardia in previous studies [3, 4]. A comparison between a chest X-ray of our patient (Fig. 4a) and a chest X-ray (Fig. 4b) of another young man of the same age group grossly demonstrated the mirror image nature of dextrocardia. In Nigeria, the incidence of dextrocardia is largely unknown but a few cases have been reported [3, 4, 6]. According to a retrospective chart review at Children’s and Women’s Health Centre of British Columbia in Vancouver, Canada, based on a population-based study of cardiac malformations and outcomes associated with dextrocardia between 1 January 1985 and 31 December 2001, the incidence of dextrocardia was estimated to be 1 in 12,019 pregnancies [10]. Dextrocardia with situs inversus may occur with other features in the respiratory tract. Kartagener syndrome occurs in approximately 25% of individuals with mirror image dextrocardia. This disorder is characterized by dextrocardia with situs inversus, sinusitis and bronchiectasis; it is associated with primary ciliary dyskinesia [2]. Our patient presented with respiratory features suggestive of Kartagener syndrome; chest X-ray findings were not remarkable and the diagnosis was not confirmed because a chest CT scan was not done due to financial constraints. Moreover, echocardiography, an investigation which may reveal an associated cardiac structural or functional lesion, was also delayed because of financial challenges. The genetic basis for dextrocardia with situs inversus is not well understood. Familial tendency to dextrocardia was described by Ibrahim in 2013 in Nigeria [11]. The type of dextrocardia associated with respiratory abnormalities was said to be autosomal recessive; however, Soltan and Li [12] described the cardiac anomaly in a kindred group in which four males were affected which suggested an X-linked recessive inheritance. Both autosomal recessive and X-linked variants have been described [13]. Because of the genetic nature of this cardiac anomaly, many unidentified cases are likely to exist among the general population which may pose diagnostic and management dilemmas to physicians because of unusual presentations at unpredictable moments [14-16]. So, screening of an apparently healthy adult population with ECG may resolve some of these medical enigmas and other asymptomatic cardiovascular disorders.

## Conclusions

A properly interpreted ECG was useful in suspecting the diagnosis of dextrocardia with situs inversus. So, analysis of a relatively simple and non-invasive diagnostic tool such as ECG allows for a suspicion of a cardiovascular anomaly in a setting of scarce diagnostic resources.

## Consent

Written informed consent was obtained from the patient for publication of this case report and any accompanying images. A copy of the written consent is available for review by the Editor-in-Chief of this journal.

## Abbreviations

CT: computed tomography
ECG: electrocardiogram
MCS: microscopy, culture and sensitivity
P wave: atrial depolarization
T wave: ventricular repolarization
